# Using Support Vector Machine and Evolutionary Profiles to Predict Antifreeze Protein Sequences

**DOI:** 10.3390/ijms13022196

**Published:** 2012-02-17

**Authors:** Xiaowei Zhao, Zhiqiang Ma, Minghao Yin

**Affiliations:** 1 College of Computer Science and Information Technology, Northeast Normal University, 2555 Jingyue Street, Changchun 130117, China; E-Mail: zhaoxw303@nenu.edu.cn; 2 Key Laboratory of Intelligent Information Processing of Jilin Universities, Northeast Normal University, Changchun 130117, China; 3 College of Life Science, Northeast Normal University, 5268 Renmin Street, Changchun 130024, China

**Keywords:** antifreeze proteins, support vector machine, position specific scoring matrix, web sever, evolutionary information

## Abstract

Antifreeze proteins (AFPs) are ice-binding proteins. Accurate identification of new AFPs is important in understanding ice-protein interactions and creating novel ice-binding domains in other proteins. In this paper, an accurate method, called AFP_PSSM, has been developed for predicting antifreeze proteins using a support vector machine (SVM) and position specific scoring matrix (PSSM) profiles. This is the first study in which evolutionary information in the form of PSSM profiles has been successfully used for predicting antifreeze proteins. Tested by 10-fold cross validation and independent test, the accuracy of the proposed method reaches 82.67% for the training dataset and 93.01% for the testing dataset, respectively. These results indicate that our predictor is a useful tool for predicting antifreeze proteins. A web server (AFP_PSSM) that implements the proposed predictor is freely available.

## 1. Introduction

Antifreeze proteins (AFPs) are functional proteins in a cell. With special antifreeze activity, AFPs make the organisms less sensitive to cold temperatures. AFPs bind to small ice crystals to inhibit growth and recrystallization of ice that would otherwise be fatal [1]. By contributing to both freeze resistance and freeze tolerance, AFPs have helped to increase species diversity in some of the harshest and most inhospitable environments. Freeze resistance involves the inactivation or removal of ice-nucleating agents in freeze-avoiding species, whereas freeze tolerance involves the activation or synthesis of ice-nucleating agents in winter in freeze-tolerant species [2,3].

AFPs have been found in various insects, fish, bacteria, fungi, and overwintering plants such as gymnosperms, ferns, monocotyledonous, angiosperms, *etc*. [4–12]. Relational analyses show that there is low sequence or structure similarity for an ice-binding domain, and lack of common features among different AFPs [7–10]. One reason for this phenomenon is that ice can present many different surfaces with different arrangements of oxygen atoms [8]. So it is difficult to establish powerful prediction methods to identify AFPs. However, AFPs play important roles in different fields, such as freeze-resistant transgenic plants and animals, food technology, preservation of cell lines, organs and cryosurgery [13,14]. How to discriminate AFPs from other proteins is important in understanding protein-ice interactions and creating new ice-binding domains in other proteins.

Many lines of evidences have indicated that computational approaches can provide useful information for both drug discovery and basic research in a timely manner [15], such as protein subcellular location prediction [16,17], structural bioinformatics [18], identification of proteases and their types [19], identification of membrane proteins and their types [20], molecular docking [21–23], identification of enzymes and their functional classes [24], and signal peptide prediction [25,26]. Up until now, there are few studies using computational approaches to discriminate AFPs and non-AFPs. Kandaswamy *et al.* [27] investigated this problem using the predictor of Random Forest. That is the first and the only method utilizing machine learning technique to deal with the prediction of AFPs. With the model AFP-Pred, they obtained 81.33% accuracy from training and 83.38% from testing. Although high accuracy has been achieved, the problem is worthy of further investigation because the performance of the aforementioned method is still not fully satisfactory and they do not provide an online web server for predicting antifreeze proteins.

In this study, we focus on developing a new antifreeze protein predictor by seeking a more informative encoding scheme. After a preliminary evaluation of different encoding schemes, we found that the evolutionary information in the form of PSSM profiles is suitable for representing the antifreeze protein sequence. Then a predictor called AFP_PSSM is established using the feature PSSM-400 as the input of support vector machine (SVM). AFP_PSSM yields 82.67% accuracy from training dataset and 93.01% accuracy from test dataset. This indicates that our predictor is very promising and may at least play an important complementary role to existing methods. The proposed predictor is freely available at the web server AFP_PSSM [28]. For a query protein sequence of 500 amino acids, it will take about 20 s for the web server to yield the predicted result; the longer the sequence is, the more time it needs.

According to a recent review [29], to establish a really useful statistical predictor, the following four procedures need to be considered: (i) construct or select a valid benchmark dataset to train and test the predictor; (ii) formulate the protein samples with an effective mathematical expression that can truly reflect their intrinsic correlation with the attribute to be predicted; (iii) introduce or develop a powerful algorithm to conduct the prediction; (iv) properly perform cross-validation tests to objectively evaluate the anticipated accuracy of the predictor; (v) establish a user-friendly web server for the predictor that is accessible to public. Below, let us describe how to cope with these procedures one by one.

## 2. Materials and Methods

### 2.1. Dataset

The datasets used in this paper is retrieved from Kandaswamy *et al.* [27] which consists of 481 antifreeze proteins and 9493 non-antifreeze proteins. To get rid of redundancy and homology bias, the sequences with ≥40% sequence similarity have been removed using program CD-HIT [30]. Then the training dataset contains 300 antifreeze proteins randomly selected from the 481 antifreeze proteins and 300 non-antifreeze proteins randomly selected from the 9493 non-antifreeze proteins. The test dataset contains the remaining 181 antifreeze proteins and 9193 non-antifreeze proteins. These datasets can be freely downloaded from [31].

### 2.2. Protein Features and Vector Encoding

To develop a powerful predictor, one of the keys is to formulate the protein sequences with an effective mathematical expression that can truly reflect their intrinsic correlation with the attribute to be predicted [17]. To realize this, some popular sequence-based encoding schemes have been investigated to represent each protein sequence.

#### 2.2.1. Evolutionary Information

Evolutionary information, one of the most important types of information in assessing functionality in biological analysis, has been successfully used to encode protein in many applications, such as our previous work of lysine ubiquitylation site prediction [32], transmembrane protein topology prediction [33] and malaria parasite mitochondrial protein prediction [34]. To extract the evolutionary information, the profile of each protein sequence is generated by running Position Specific Iterated BLAST (PSI-BLAST) program [35,36]. Then this information can be represented as a two dimensional matrix which is known as the PSSM of the protein.

In this paper, the PSSM of each protein sequence in the constructed dataset is generated against the non-redundant Swiss-Prot database [37] (version 56, released on 22 July, 2008) using the PSI-BLAST program with three iterations (−j 3) and e-value threshold 0.0001 (−h 0.0001). This matrix is composed of L × 20 elements, where L is the total number of residues in a peptide. The rows of the matrix represent the protein residues and the columns of the matrix represent the 20 naive amino acids. Each element represents the probability of the occurrence of each 20 amino acid when it’s mutated to the others at one position during the evolution process.

**Figure 1:**
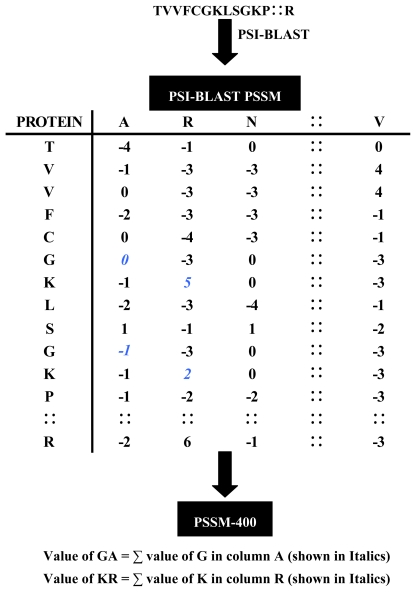
Schematic representation of transformation each protein sequence into PSSM-400 matrix.

In the view of the fact that SVM requires the fixed length feature vectors as their inputs for training, we generate a vector of dimension 400, called PSSM-400 from the PSSM. PSSM-400 is composition of occurrences of each type of amino acid corresponding to each type of amino acids in protein sequence [38]. Thus for each column we have a vector of dimension 20. Figure 1 shows the schematic representation of transformation each protein sequence into PSSM-400.

#### 2.2.2. Amino Acid and Dipeptide Composition

The purpose of calculating composition of proteins is to transform the variable length of protein sequence into fixed length feature vectors [33]. This is a necessary step during classification of proteins using SVM. The transformation of each protein sequence into a vector of 20 dimensions using amino acid composition will encapsulate the information of protein. Besides amino acid composition, dipeptide composition is also utilized, which gives a fixed pattern length of 400. The advantage of dipeptide composition compared with amino acid composition is that it encapsulates both the fraction information of amino acids and the local order information of protein sequence.

#### 2.2.3. Chou’s Pseudo Amino acid Composition

The Chou’s pseudo amino acid composition (PseAAC) encoding scheme feature has been widely used to predict various properties of proteins [39–43]. It can be calculated as following:

(1) $$PseAAC(i)=\left\{ \begin{array}{l} \frac{f(i)}{\sum_{j=1}^{20}f(j)+\omega \sum_{j=1}^{30}\theta (j)},i=1,2,\ldots ,20,\\ \frac{\omega \cdot \theta (i-20)}{\sum_{j=1}^{20}f(j)+\omega \sum_{j=1}^{30}\theta (j)},i=21,22,\ldots ,50 \end{array}\right.$$

(2) $$\theta (d)=\frac{1}{L-d}\sum_{i=1}^{L-d}\Theta (d_{i,i+d}),d=1,2,\ldots ,30$$

Where *ω* is a weighting factor (default *ω* = 0.1). $\Theta (d_{i,i+d})=\frac{1}{3}\sum_{j=1}^{3}{\left(H_{j,i}-H_{j,i+d}\right)}^{2}$, *H*_1_,*_i_*, *H*_2_,*_i_*, and *H*_3,_*_i_*, are the three amino acid properties [44] in Table S1 (see Supplementary Material). It’s obvious that there are 50 features generated from Chou’s pseudo amino acid composition.

### 2.3. Support Vector Machines

Support vector machine (SVM) [45] belongs to the family of margin-based classifier and is assumed to be a very powerful method to deal with prediction, classification, and regression problems. SVM look for optimal hyperplane which maximizes the distance between the hyperplane and the nearest samples from each of the two classes. Formally, given a training vector x*_i_* ε R^n^ and their class values y*_i_* ε {−1, 1}, *i* = 1, …, *N*, SVM solve the following optimization problems:

(3) $$\text{Minimize}\frac{1}{2}w^{T}\cdot w+C\sum_{i=1}^{N}\xi_{i}$$

(4) $$\text{Subject to }y_{i}(w^{T}\cdot x_{i}+b)\geq 1-\xi_{i}\;\text{and }\xi_{i}\geq 0$$

where *w* is a normal vector perpendicular to the hyperplane and *ξ_i_* are slake variables for allowing misclassifications. Here *C* (>0) is the penalty parameter which balances the trade-off between the margin and the training error. In this study, LIBSVM package [46,47] with radial basis kernel function is used. Two parameters, the regularization parameter *C* and the kernel width parameter γ are optimized based on 5-fold cross-validation using a grid search strategy.

### 2.4. Evaluation

Ten-fold cross validation [48] is used in this work. The dataset is randomly divided into ten equal sets, out of which nine sets are used for training and the remaining one for testing. This procedure is repeated ten times and the final prediction result is the average accuracy of the ten testing sets. To reduce the computational time, we also adopt the independent testing dataset cross validation in this study as done by [49] to evaluate our model.

Three parameters, sensitivity (*S_n_*), specificity (*S_p_*), and accuracy (*Acc*) are used to measure the performance of our model. They are defined by the following formulas:

(5) $$S_{n}=\frac{TP}{TP+FN}\times 100$$

(6) $$S_{p}=\frac{TN}{TN+FP}\times 100$$

(7) $$Acc=\frac{TP+TN}{TP+TN+FP+FN}\times 100$$

where *TP*, *TN*, *FP* and *FN* stand for true positive, true negative, false positive and false negative, respectively. Moreover, we create ROC (receiver operating curve) for all of the models in order to evaluate the performance of models using different encoding schemes.

### 2.5. Model Building and Protocol Guide

**Figure 2:**
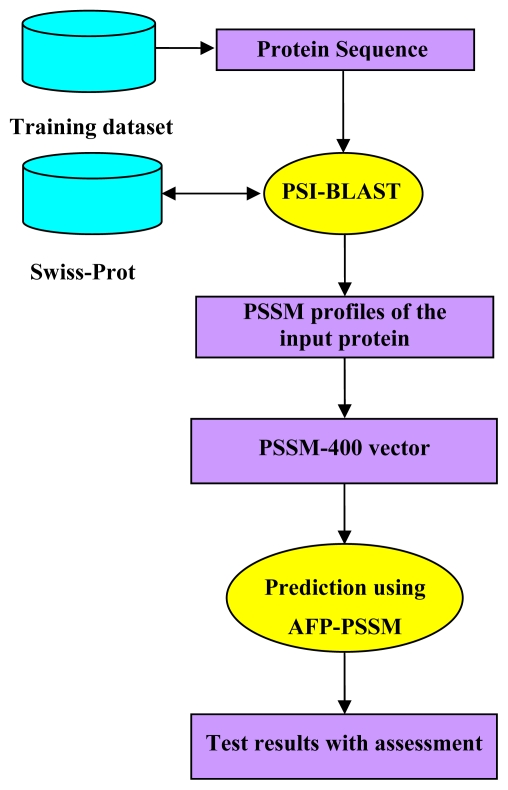
The workflow of the AFP_PSSM predictor.

The detailed flowchart of our work is shown in Figure 2. First, sequential evolution information in form of PSSM profiles for the input sequence is generated by PSI-BLAST. Second, the obtained PSSM is further transformed into PSSM-400 vector. Finally, the predictor AFP_PSSM is applied to output the test results.

**Figure 3:**
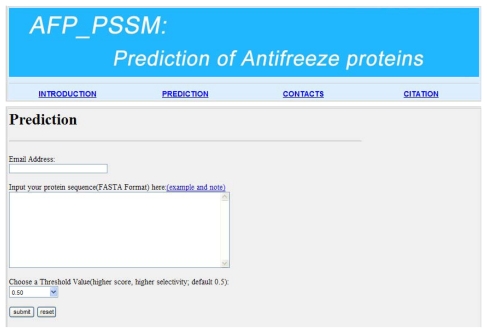
The top page of the AFP_PSSM web server [28].

**Figure 4:**
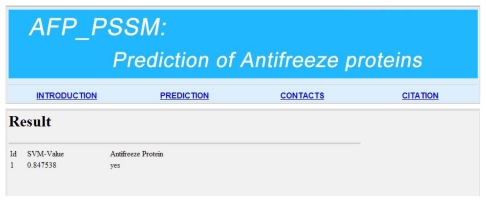
The prediction results by AFP-PSSM for the query protein 1 in the example and note window.

For the convenience of experimental scientists, we give a step-by-step guide on how to use it to get the desired results as follows: (i) Open the web server AFP_PSSM [28] and you can see the prediction page on your computer screen, as shown in Figure 3. You must input your email address since the prediction process may take a long time; (ii) Input your query protein sequence to the text box in Figure 3. Note that the input protein sequence should be in the FASTA format. The FASTA format sequence consists of a single initial line beginning with a greater-than symbol (“>”), followed by lines of amino acid sequence. You can click on the “example and note” button to see the example protein sequence; (iii) Choose a threshold value in the drop-down list. For prediction with high confidence (less probability of false positive prediction), high threshold should be chosen; (iv) Click on the submit button to see the predicted result. For example, if you use the first sequence in the example page, the predicted result will be “0.847538, yes” as can be seen in Figure 4, which means that the protein is an antifreeze protein with the probability of 0.847538. It takes about 15 s for a protein sequence of 300 amino acids before the predicted result appears.

## 3. Results and Discussion

**Figure 5:**
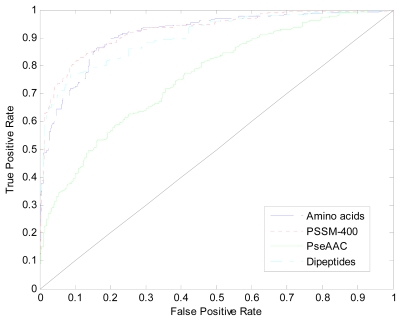
The receiver operating characteristic (ROC) curves calculated from the ten-fold cross validation of the four different models.

In this section, four SVM models based on amino acids composition, dipeptides composition, Chou’s PseAAC and PSSM-400 are constructed respectively. The accuracies and receiver operating characteristic (ROC) curves for these four SVM models are shown in Table 1 and Figure 5. One can see that PSSM-400 encoding scheme performs better than the others with accuracy of 82.67% and AUC (Area Under Curve) of 0.926. Thus we use it as our final encoding scheme to represent antifreeze protein sequences.

In order to further examine the prediction of power of the current classifier, we compare our predictor AFP_PSSM with the recent work of Kandaswamy *et al.* [27] on the testing dataset. The number of antifreeze proteins and non-antifreeze proteins in the testing dataset are highly imbalanced, and this situation is close to reality. The compared results are shown in Table 2. As can be seen from the table, the predictor proposed in this study obtains accuracy of 90.17%, higher than the accuracy of 83.38% gained by [27]. The better prediction performance may be credited to the appropriate protein sequence encoding scheme adopted in our prediction model.

## 4. Conclusions

Accurate identification of new antifreeze proteins is important in understanding ice-protein interactions and creating novel ice-binding domains in other proteins. Though some researchers have focused on this problem, the accuracy of prediction is still not satisfied, and there are few online web servers for predicting antifreeze protein sequences. In this paper, a highly accurate method is developed for predicting antifreeze proteins using support vector machine and evolutional profiles. This is the first paper in which evolutionary information in the form of PSSM profiles has been utilized to predict antifreeze proteins. The proposed predictor is freely available at the web serve AFP_PSSM [28].

## Supplementary Information



## Figures and Tables

**Table 1:** The accuracies and Area Under Curve (AUC) of the four support vector machine (SVM) models developed using different features. These models are trained and tested on the training dataset.

Method	Amino Acids	Dipeptides	PseAAC	PSSM-400
Acc	80.83%	78.83%	56.18%	82.67%
AUC	0.912	0.904	0.761	0.926

**Table 2:** Comparison with AFP-Pred on the test dataset.

Method	S_n_ (%)	S_p_ (%)	Acc (%)
AFP-Pred [27]	84.67	82.32	83.38
AFP_PSSM	75.89	93.28	93.01
