# Supplementary material for: Using Support Vector Machine and Evolutionary Profiles to Predict Antifreeze Protein Sequences
Source: Int J Mol Sci. 2012 Feb 17;13(2):2196–207. doi: 10.3390/ijms13022196 (PMC3292016; doi:10.3390/ijms13022196)
Supplement: Supplementary file 1 [file ijms-13-02196-s001.pdf]

Supplementary Information

Table S1. The physicochemical properties of the amino acids.

Order	Physicochemical Property	Range of Property
1	Hydrophobicity	[−2.53, 1.38]
2	Hydrophilicity	[−3.4, 3]
3	Side-chain mass	[1, 130]

© 2012 by the authors; licensee MDPI, Basel, Switzerland. This article is an open access article distributed under the terms and conditions of the Creative Commons Attribution license (http://creativecommons.org/licenses/by/3.0/).
